# A Systematic Review of the Role of Recreational Activities and Dietary Management in Treating Hypertension and Hyperglycaemia in Adults in Low- and Middle-Income Countries

**DOI:** 10.3390/ijerph23080958

**Published:** 2026-07-24

**Authors:** Khutso Lekgothoane, Selekane Ananias Motadi, Osia Livhuwani Munyangane

**Affiliations:** 1 South African Recreation and Leisure Professionals Association, Potchefstroom 2531, South Africa; 2 Department of Nutrition, University of Venda, Thohoyandou 0950, South Africa; selekane.motadi@univen.ac.za; 3 Department of Indigenous Knowledge Systems, University of Venda, Thohoyandou 0950, South Africa; livhuwaniosia@gmail.com

**Keywords:** active recreational activities, dietary management, hyperglycaemia, hypertension

## Abstract

**Highlights:**

**Public health relevance—How does this work relate to a public health issue?**

- Considering the socio-economic status of people in low- and middle-income countries, this study recommends affordable, preventive multi-component intervention strategies, including lifestyle modifications, therapeutic recreation, and dietary management.
- Integrating therapeutic recreation and diet into community-based programmes encourages sustainable behavioural changes and improves adherence to treatment plans.

**Public health significance—Why is this work of significance to public health?**

- It contributes to the economics of knowledge in alternative multi-component approaches to controlling diseases within the healthcare ecosystem in low- and middle-income countries.
- It creates awareness, empowering patients to self-manage their conditions and reducing the pressure on overstretched health systems, ultimately improving health outcomes and reducing inequalities in low-resource settings.

**Public health implications—What are the key implications or messages for practitioners, policy makers and/or researchers in public health?**

- Since this multi-component self-management approach is recommended, it will help lower healthcare burdens and costs in the healthcare ecosystem in low- and middle-income countries.
- These recommended approaches will improve population health and strengthen prevention-focused healthcare systems in low- and middle-income countries.

**Abstract:**

In low- and middle-income countries, the burden of hypertension and hyperglycaemia is alarming. These conditions remain major, recurring public health concerns that demand alternative healthcare strategies. This systematic review critiques and synthesises the existing evidence on the roles of recreational activities and dietary management in treating hypertension and hyperglycaemia in adults in low- and middle-income countries. The aim is to create awareness for hypertensive and hyperglycaemic patients. A systematic search was conducted using the PRISMA guidelines across six online databases: PubMed, Scopus, Google Scholar, Cochrane Library, Web of Science, and CINAHL. The search included peer-reviewed articles published between 2015 and 2025. Of the 12,500 identified articles, only 35 met the inclusion criteria. The evidence suggests that active recreational activities and dietary interventions are the most effective strategies for managing hypertension and hyperglycaemia when implemented properly. Current studies demonstrate that aerobic recreational activity improves vascular function, lowers blood pressure, and enhances insulin sensitivity. DASH and Mediterranean diets support cardiometabolic health in hypertensive and hyperglycaemic patients. These diets help reduce hypertension, control blood sugar levels, and lower cardiovascular risk. If sustained, these intervention strategies can effectively prevent and manage hypertension and hyperglycaemia. This review recommends that healthcare systems integrate therapeutic recreational activities with the DASH and Mediterranean diets as alternative interventions for adults with hypertension and hyperglycaemia.

## 1. Introduction

In low- and middle-income countries (LMICs), the burden of hypertension and hyperglycaemia is alarming and remains a major, recurring public health concern [1,2]. These concerns demand alternative responsive healthcare strategies. Because a significant proportion of both inpatients and outpatients are diagnosed with one or both conditions, this puts pressure on health systems that are already constrained by resource shortages. Many healthcare centres in most of these countries rely primarily on pharmaceutical care as a key strategy for managing hypertension and hyperglycaemia. This could be because their healthcare is shaped by the limited resources within their facilities. Shortages of trained staff and inconsistent availability of diagnostic tools are common challenges in most LMIC facilities. This problem compromises effective disease control, and these conditions require multiple intervention strategies.

Hypertension and hyperglycaemia are closely interrelated non-communicable diseases (NCDs) that often result from being overweight and physically inactive. When both conditions occur in a patient, they increase the risk of cardiovascular and renal complications if not managed early [3]. Renal complications may require dialysis, and in most public hospitals, dialysis units are often fully booked. Given limited resources and the serious risks associated with these conditions, prevention and management are essential for patients with hypertension and hyperglycaemia. Scientific evidence strongly supports food-based dietary interventions for managing these conditions.

The main point is that both the DASH and Mediterranean diets can improve blood pressure, glycaemic control, and overall cardiometabolic health, but their effectiveness depends on long-term adherence. The DASH diet is particularly effective when sustained, while the Mediterranean diet shows long-term benefits for insulin sensitivity and cardiovascular health [4,5,6,7,8].

The main argument is that combining dietary approaches with regular recreational aerobic and resistance exercise provides a complementary strategy. This joint approach reduces vascular resistance, improves insulin action, and lowers circulating glucose [4,6,9]. Adopting a healthy lifestyle that sustains balanced diets and activity is a cost-effective, sustainable intervention, especially beneficial for helping patients in resource-constrained settings to manage their conditions.

The main argument of this review is that an integrated approach combining pharmacological care, lifestyle modifications like diet and exercise, early screening, public health education, and community interventions provides an effective and sustainable solution to the dual burden of hypertension and hyperglycaemia in LMICs. This strategy can alleviate strain on health systems, prevent economic threats, and significantly improve health outcomes for adults worldwide.

### Objective of the Study

This systematic review critiques and synthesises existing evidence on the role of recreational activities and dietary management in treating hypertension and hyperglycaemia in adults in low- and middle-income countries in order to raise awareness among hypertensive and hyperglycaemic patients.

## 2. Materials and Methods

### 2.1. Study Design

A systematic review was conducted in accordance with PRISMA 2020 recommendations. The research question was formulated in line with the study’s overall purpose. To develop a focused, structured question, the authors of this review used the PICO framework (Population, Intervention, Comparator, Outcome). Using this approach, the guiding question was framed as follows: in what way do recreational activities and dietary strategies contribute to the management of hypertension and hyperglycaemia among adults in LMICs?

### 2.2. Review Question

This systematic review aimed to answer the following question:

How do recreational activities and dietary interventions (DASH and Mediterranean diets) manage hypertension and hyperglycaemia among adults?

To guide the development of this review question and eligibility criteria, the PICO framework was applied:

- Population (P): Adults (≥18 years) diagnosed with hypertension, hyperglycaemia, or type 2 diabetes.
- Intervention (I): Therapeutic recreational activities (e.g., walking, cycling, swimming, dancing, structured exercise) and dietary interventions (DASH diet and Mediterranean diet).
- Comparison (C): Standard healthcare, usual lifestyle practices, or alternative interventions.
- Outcomes (O): Clinical outcomes such as blood pressure, fasting glucose, HbA1c, insulin sensitivity, BMI, lipid profile, and overall cardiometabolic health.

The framework provided a structured, focused basis for reviewing the available evidence on lifestyle-based strategies for managing hypertension and hyperglycaemia across diverse adult populations.

### 2.3. Protocol and Registration

The review protocol was registered with PROSPERO to promote transparency and avoid duplication (Registration Number: CRD420251161063). The registration outlines the review objectives, criteria, search strategy, and data synthesis methods. This step follows best practices for systematic reviews and ensures accountability.

### 2.4. Inclusion and Exclusion Criteria

The PICO framework was used to identify studies based on specific inclusion and exclusion criteria as presented in Table 1.

Inclusion criteria for this systematic review were carefully designed. To ensure that only relevant, high-quality studies addressing the management of hypertension and hyperglycaemia are included. This review focused on patients aged 18 years or older and those at high risk of cardiometabolic complications.

### 2.5. Search Strategy

The search strategy employed a combination of keywords and Boolean operators to maximise coverage and capture all pertinent literature.

The following search terms were used in combination with appropriate Boolean operators:

(“active recreational activities” OR “physical activity” OR exercise) AND (“ dietary” OR “DASH diet” OR “Mediterranean diet” OR “dietary intervention”) AND (hyperglycaemia OR “high blood glucose” OR “type 2 diabetes”) AND (hypertension OR “high blood pressure”). Additional filters were applied: abstracts, full texts, clinical trials, reviews, human subjects, and age ≥18 years. The search strategy was adapted for each database.

### 2.6. Study Selection Process

Two reviewers (KL and SAM) independently screened titles and abstracts using Rayyan software (version 1.7.7) to identify studies. Each record was first assessed against predefined inclusion and exclusion criteria (Table 1) by both KL and SAM. If KL and SAM disagreed, a third reviewer (OLM) then reviewed the record and made a final decision. This process ensured consensus and objectivity, aligned with best practices in systematic review methods, and minimised bias while strengthening inter-rater reliability.

Following this stage, KL, SAM, and OLM retrieved and assessed the full texts of potentially relevant articles to determine their eligibility. Studies that met all inclusion criteria were retained, while those that failed to meet methodological or outcome requirements were excluded. To further ensure methodological rigour, any remaining uncertainties were resolved in consultation with a third reviewer (OLM).

### 2.7. Data Extraction

Two reviewers (KL and SAM) independently extracted data using a standardised form developed for this review. After completing their extractions, the reviewers compared results to identify and address discrepancies through discussion. If they could not reach a consensus, a third reviewer (OLM) adjudicated and made the final decision. Extracted information included study characteristics (author, year, country, study design, sample size, and population), intervention details (type, duration, and intensity of recreational activity or dietary programme), comparator, and reported outcomes. These outcomes included blood pressure, glycaemic control, insulin sensitivity, BMI, lipid profiles, and other cardiometabolic indicators.

### 2.8. Quality Assessment

The methodological quality of included studies was independently appraised by two reviewers (OLM and SAM). Randomised controlled trials were assessed using the Cochrane Risk of Bias Tool. At the same time, observational and cohort studies were evaluated using the Newcastle–Ottawa Scale, and reviews and meta-analyses were evaluated using the AMSTAR-2 checklist. Each study was rated as low, moderate, or high quality. Disagreements were resolved through discussion or consultation with a third reviewer (SAM). Risk of bias assessments guided the interpretation of findings and highlighted methodological limitations in the evidence base.

### 2.9. Data Synthesis

A narrative synthesis was employed due to heterogeneity in study designs, interventions, and outcome measures. To provide structure, findings were grouped into two main categories: (1) recreational activities and (2) dietary interventions (DASH and Mediterranean diets). Within each category, evidence was synthesised to highlight effects on blood pressure, glycaemic control, and broader cardiometabolic health. Additionally, where available, data were further compared by study setting (high-income vs. low- and middle-income countries) to assess contextual differences. Throughout this synthesis, consistent patterns of evidence were highlighted, and gaps in the literature were identified to inform future research.

### 2.10. Ethical Considerations

This was a systematic review, and no primary data involving human participants were collected. Therefore, ethical approval was not required.

## 3. Results

### 3.1. Screening and Selection Process

**Figure 1:**
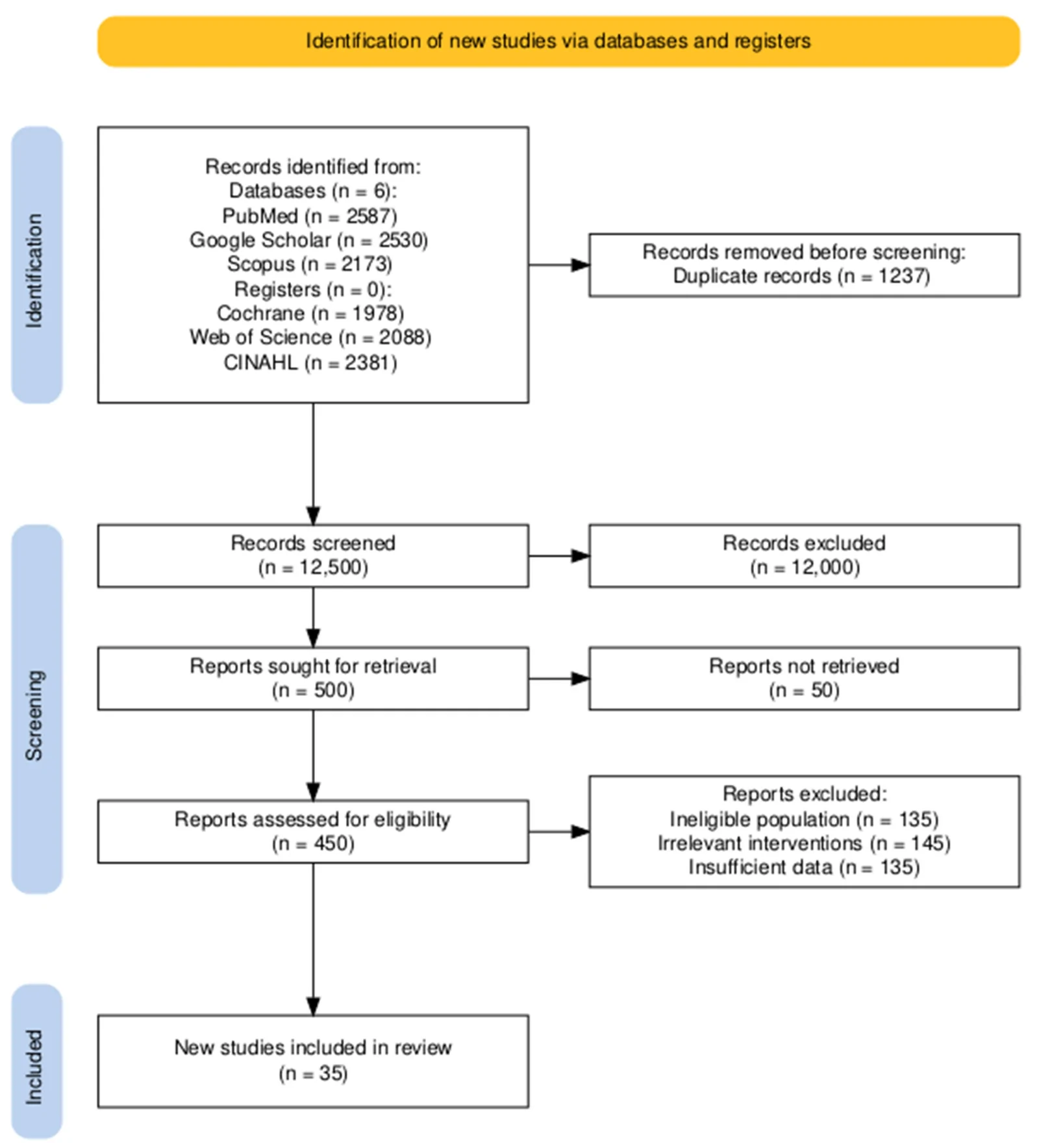
PRISMA flowchart [10].

The screening and selection process for this systematic review was conducted systematically. The main reason for this was to ensure the inclusion of relevant, high-quality studies. A comprehensive search across multiple databases, including PubMed, Scopus, Web of Science, CINAHL, and the Cochrane Library, as well as grey literature, was conducted to identify potential studies. Titles and abstracts were screened against predefined inclusion and exclusion criteria. The duplicates were removed. The ineligible studies were excluded. Full-text articles were then assessed for methodological quality, relevance of interventions, and outcome measures, with discrepancies resolved through discussion among reviewers. Ultimately, the studies included in the final synthesis are illustrated in the PRISMA flow diagram below (Figure 1).

### 3.2. Study Characteristics

A total of 35 studies met the inclusion criteria for this systematic review. The included studies were conducted across diverse settings, including high-income countries such as the United States, Canada, Australia, and several European nations [11,12,13,14], as well as low- and middle-income countries (LMICs) such as India, Nepal, Kenya, Iran, and China [6,15,16,17]. This diversity [6,15,16] was included for cross-contextual comparisons between resource-rich and resource-limited environments.

Study designs varied, with the majority comprising randomised controlled trials [6,11,18,19], cohort studies [20], and intervention-based trials [21,22], while several were systematic or narrative reviews providing secondary evidence [2,4,23]. Sample sizes ranged from small community-based studies to large multinational cohorts [24,25].

Interventions fell into two main categories:

1. Recreational activities: Several studies examined walking, cycling, swimming, yoga, resistance training, or structured exercise programmes, all demonstrating positive effects on hypertension and glycaemic regulation [16,20,21]. Some interventions combined dietary and physical activity strategies, showing synergistic benefits [11,26].
2. Dietary interventions take centre stage in most studies, with the DASH and Mediterranean diets leading to significant benefits in blood pressure, glycaemic control, and lipid profiles [13,27]. Importantly, a few studies introduced creative variations, such as integrating time-restricted eating with DASH [6] or tailoring diets to specific groups, demonstrating innovation and adaptability.

Comparators in these studies varied: some included standard care, others assessed usual lifestyle practices, and others compared alternative non-structured interventions. This approach enabled a clearer understanding of the added value of structured diet and activity-based interventions [15,17].

Researchers assessed outcomes such as systolic and diastolic blood pressure, fasting glucose, HbA1c, insulin sensitivity, BMI, waist circumference, and lipid profiles. Most interventions enhanced vascular function, metabolic regulation, and overall cardiometabolic health through recreational activities and dietary strategies [4,9].

Regarding geographic distribution, the majority of studies were conducted in high-income countries, with fewer contributions from African and other LMIC contexts [2,15]. This represents a critical evidence gap, as feasibility, cultural acceptability, and adherence may differ significantly across populations.

In summary, evidence from the included studies consistently supports the effectiveness of recreational activity and dietary modification in managing hypertension and hyperglycaemia. Nevertheless, direct comparability was limited by heterogeneity in design, intervention duration, and outcome reporting [23,28].

The findings in Table 2 reveal several management strategies for hypertension and hyperglycaemia. Notably, most USA studies reported the DASH and Mediterranean diets as central intervention strategies. Both diets provide robust evidence of efficacy, though their mechanisms and areas of greatest impact differ. Specifically, the DASH diet offers pronounced reductions in blood pressure, while the Mediterranean diet helps to control glycaemia. Additionally, some studies indicated that recreational activities improve vascular function and enhance insulin sensitivity. Other studies have emphasised the importance of integrating recreational activities with DASH and Mediterranean diets.

### 3.3. Intervention Types

#### 3.3.1. Dietary Interventions (DASH and Mediterranean Diets)

The majority of included studies evaluated dietary interventions, particularly the DASH and Mediterranean diets. Across the reviewed literature, adherence to the Dietary Approaches to Stop Hypertension (DASH) eating pattern was consistently associated with meaningful reductions in blood pressure [40]. Reported decreases in systolic blood pressure generally ranged between 5 and 11 mmHg, while diastolic pressure declined by approximately 3 to 6 mmHg [6]. In addition to these effects, some studies reported slight improvements in glycaemic control, with HbA1c reductions typically ranging from 0.3% to 0.8%, influenced by participants’ baseline metabolic status and the duration of the intervention, which commonly spanned 8 to 24 weeks.

In comparison, the Mediterranean dietary pattern appeared to exert more pronounced effects on glycaemic outcomes. Studies have frequently documented HbA1c reductions of 0.5% to 1.0%, along with enhanced insulin sensitivity and improved lipid profiles [14]. Although reductions in blood pressure were also observed, these were generally less substantial than those reported for DASH-based interventions. Intervention periods varied from approximately three months to over one year, with evidence suggesting that longer adherence contributed to greater overall cardiometabolic improvements.

#### 3.3.2. Active Recreational Activity Interventions

Studies examining active recreational activities primarily focused on aerobic-based exercises such as walking, cycling, swimming, and structured exercise programmes. These interventions were associated with moderate reductions in blood pressure, with systolic values decreasing by approximately 4–9 mmHg and diastolic values by approximately 2–5 mmHg [21,36]. Improvements in glycaemic control were also observed, particularly among individuals with type 2 diabetes, with HbA1c levels declining by 0.4% to 0.9%. The duration of interventions ranged from 8 weeks to 12 months, with evidence indicating that programmes of greater intensity and longer duration yielded stronger outcomes.

Furthermore, several studies have documented additional health benefits, including enhanced vascular function, improved insulin sensitivity, and reduced body mass index (BMI), underscoring the broad, multifaceted impact of recreational physical activity.

#### 3.3.3. Combined Lifestyle Interventions (Diet + Physical Activity)

Only a limited number of studies examined interventions that combined dietary changes with physical activity. However, these multi-component strategies produced the most substantial overall benefits, with reductions in systolic blood pressure often surpassing 10 mmHg and decreases in HbA1c reaching or exceeding 1.0% in certain populations [26]. Despite these promising outcomes, multi-component strategies and integrated lifestyle approaches remain relatively scarce in the existing literature, highlighting a notable gap in research on comprehensive interventions.

### 3.4. Comparative Effectiveness and Consistency of Findings

The evidence indicates consistent positive effects across different types of interventions, although outcomes vary depending on study design, intervention intensity, and participant characteristics. Dietary strategies tended to have a greater influence on long-term metabolic outcomes, whereas physical activity interventions were associated with a wider range of physiological benefits [11]. Interventions that combined both approaches yielded the most clinically significant improvements, despite being examined in relatively few studies.

### 3.5. Quality and Bias Assessment

The quality of the included studies ranged from moderate to high, with most evidence derived from randomised controlled trials and cohort studies. These studies generally support the effectiveness of active recreational activities and dietary interventions. Several biases were noted. Selection bias was common due to convenience sampling. Therefore, adherence to interventions was often self-reported, introducing performance bias. Attrition in long-term studies and variations in outcome measurement contributed to potential detection bias.

Additionally, publication bias may have influenced the evidence base. Studies reporting positive outcomes are more likely to be published. Despite these limitations, the overall findings provide a reliable indication of the benefits of lifestyle interventions, though caution is needed in interpreting the results across diverse populations and settings.

## 4. Discussion

This systematic review synthesised existing evidence on the role of active recreational activities in the management of hypertension and hyperglycaemia to raise awareness among adults. The findings suggest that active participation in a combination of recreational activities that have aerobic and resistance training can lower blood pressure and improve glycaemic control in patients [41]. Scientific evidence consistently demonstrates that engaging in regular aerobic-based recreational activities reduces both systolic and diastolic blood pressure. Aerobic recreational activities such as brisk walking, swimming, and dancing have been recognised as appropriate for improving vascular elasticity, reducing arterial stiffness, and enhancing endothelial function in hypertensive and hyperglycaemic patients.

The literature indicates that repeated bouts of active recreational activities increase blood flow, generating shear stress along the arterial walls of hypertensive and hyperglycaemic patients [42]. This mechanical stimulus promotes favourable structural changes, such as the preservation of elastin and a reduction in collagen deposition. As a result, arteries become more compliant, allowing them to expand and recoil efficiently. In hypertensive individuals, this improved elasticity contributes to reduced peripheral resistance and lower resting blood pressure. However, these manifest only when appropriately administered in a home-based or clinical setting. Structured recreational activity also supports cardiovascular health and weight management, which are critical for the prevention and control of hypertension.

The positive effects of active recreational activity on hyperglycaemia are well established. Studies consistently show that regular light- to moderate-intensity active recreational activities improve insulin sensitivity in hypertensive and hyperglycaemic patients [28,43]. They are scientifically proven to boost skeletal muscle glucose uptake and lower glycated haemoglobin (HbA1c) in patients with type 2 diabetes [9,31]. These benefits are largely mediated through physiological mechanisms. Active recreational activities stimulate muscle contractions, which promote insulin-independent glucose uptake by increasing the translocation of GLUT4 transporters to the cell membrane [44,45], while also enhancing insulin signalling pathways and receptor sensitivity in the post-exercise period. In addition, regular participation in active recreational activities improves mitochondrial function, reduces adiposity, and lowers chronic inflammation. This contributes to more efficient insulin action and glucose metabolism.

In addition to active recreational activities. Dietary interventions are fundamental to managing hypertension and hyperglycaemia [40]. The literature underscores the importance of dietary interventions that include consumption of fruits, vegetables, whole grains, legumes, nuts, lean protein, and low-fat dairy [40,46]. These are essential elements of the DASH intervention. Fruits provide key nutrients that directly support blood pressure control and glucose regulation in hypertension and hyperglycaemia patients [47]. Research demonstrates that fruits are rich sources of potassium, a mineral that counteracts the effects of sodium [47]. Elevated sodium intake is a significant risk factor for hypertension [39,48]. Potassium contributes to the relaxation of blood vessel walls, thereby improving vascular tone and reducing blood pressure. For optimal hypertension management, adherence to the DASH diet is recommended.

Nutritionally, the literature shows that the DASH diet is rich in the following nutrients that patients following DASH should consume potassium, magnesium, calcium, and dietary fibre which regulate vascular tone and improve insulin sensitivity in individuals with hypertension [40]. Patients should adhere to this diet consistently to achieve positive outcomes. Evidence consistently demonstrates its efficacy in reducing blood pressure [26,27,33], while additional studies have confirmed benefits for fasting glucose and lipid profiles in patients with type 2 diabetes. However, it is worth highlighting that adherence remains a dietary challenge among hypertensive and hyperglycaemic patients. This may be because patients are exposed to processed and packaged foods in their communities.

Another dietary pattern of significant relevance is the Mediterranean diet, which should be considered or recommended by health practitioners for patients [34]. This diet strongly recommends consuming fruits, vegetables, and olive oil as the primary sources of fat, along with moderate fish and poultry, moderate wine intake, and limited red meat intake [23,27,35], unlike the DASH diet, which is designed primarily to prevent hypertension. Mediterranean diet reduces cardiovascular morbidity and improves glycaemic control, making it particularly beneficial in populations with a high prevalence of diabetes. This review suggests integration of diet with recreational activity. This integration is cost-effective and sustainable when administered at home rather than in a clinical setting. Hypertensive and hyperglycaemic patients should adopt this non-pharmacological approach.

## 5. Strengths and Limitations of the Review

This systematic review shows several strengths. A broad, structured search across multiple databases increased the likelihood of finding relevant studies. Including randomised controlled trials, cohort studies, and review articles offered a comprehensive perspective. Following PRISMA guidelines promoted transparency and reproducibility. Despite its strengths, this review has limitations. The included studies varied in intervention approaches, duration, outcome measures, and participant characteristics, making direct comparison impossible and meta-analysis unfeasible. Most studies were conducted in high-income settings, creating a geographic imbalance and reducing their relevance to low- and middle-income regions. These factors should be taken into account when interpreting the results. Future research would benefit from standardised reporting of outcomes, increased representation of under-researched regions, and rigorous study designs that minimise bias. Additionally, investigating the effectiveness of interventions in low- and middle-income contexts and utilising objective measures of adherence would help address current gaps.

## 6. Conclusions

This systematic review highlights that hypertension and hyperglycaemia frequently coexist in adults. Both conditions remain major public health challenges in LMICs. Regular participation in activities such as walking, cycling, swimming, and dancing is beneficial, and it achieves the following:

▪ Improves the vascular function of the participants;
▪ Enhances the insulin sensitivity of the participants;
▪ Reduces the body weight of the participants;
▪ Lowers the blood pressure of the participants;
▪ Circulates the glucose levels of the participants.

However, to achieve the optimal health outcomes, long-term adherence to the programmes is highly recommended for patients.

Dietary interventions also play a crucial role in managing hypertension and hyperglycaemia in adults. Patients should follow the DASH eating plan, which recommends a 2000-calorie-a-day diet. This plan helps lower blood pressure and improve cardiometabolic outcomes. The diet includes a variety of vegetables, whole grains, and reduced sodium intake. It demonstrates significant effectiveness.

Equally important as the above, the Mediterranean diet, centred on plant-based foods, olive oil, legumes, fish, and moderate wine intake, has been shown to benefit glycaemic control, cardiovascular health, and longevity. This diet’s daily recommendations are:

▪ To eat 5 servings of vegetables (leafy greens and tomatoes).
▪ To eat 2–3 servings of fruits.
▪ To eat 4–6 servings of whole-grain bread, pasta, or brown rice.
▪ To drink 2–4 tablespoons of Extra Virgin Olive Oil per day as the main source of added fat.

Importantly, neither dietary change nor recreational activity alone is sufficient to address the complex pathophysiology of hypertension and hyperglycaemia fully. Rather, a synergistic approach that combines structured recreational activity with adherence to healthy dietary patterns offers the most sustainable and effective management strategy for these conditions.

In LMICs, where the burden of these conditions is increasing rapidly, integrating lifestyle interventions into public health initiatives is strategically important. The primary approach is early community screening and disclosing negative health outcomes from medical screenings. Next, diet plans should be tailored to cultural and socio-economic factors. Third, community- and home-based recreational initiatives should be promoted to manage these conditions. Based on the discussed health outcomes and intervention strategies, further studies are required to identify effective combinations of therapeutic recreational activities and diet.

## Figures and Tables

**Table 1:** Inclusion and exclusion criteria that guided this systematic review.

Criteria Type	Criteria	Description/Rationale
**Inclusion**	**Population**	Adults (≥18 years) diagnosed with hypertension, hyperglycaemia, or type 2 diabetes, including overweight or obese adults in LMICs
	**Intervention**	Therapeutic recreational activities may include walking, cycling, swimming, dancing, or structured exercise; dietary interventions may involve DASH diet, Mediterranean diet, or other structured dietary programmes
	**Comparison**	Standard care, usual lifestyle, or alternative interventions allow assessment against typical management
	**Outcomes**	Measured blood pressure, fasting glucose, HbA1c, insulin sensitivity, BMI, waist circumference, lipid profiles, or other cardiometabolic risk indicators; focuses on clinically meaningful outcomes
	**Study Design**	Randomised controlled trials (RCTs), quasi-experimental studies, cohort studies, and reviews/meta-analyses
	**Language**	English
	**Publication Date**	Last 10 years; ensures contemporary relevance and applicability
**Exclusion**	**Population**	Children, adolescents (<18 years), pregnant women, or populations without diagnosed hypertension or hyperglycaemia; ensures focus on adult populations at risk for cardiometabolic disease
	**Intervention**	Pharmacological-only interventions, unstructured or unspecified lifestyle interventions, or studies without recreational activity or dietary components; focuses on lifestyle-based management
	**Study Design**	Case reports, editorials, commentaries, letters, conference abstracts without full text, or studies with insufficient methodological detail; ensures inclusion of high-quality, reliable evidence
	**Outcomes**	Studies that do not report blood pressure, glycaemic measures, or relevant cardiometabolic outcomes; ensures focus on measurable, clinically relevant endpoints
	**Language**	Non-English publications with no English translation
	**Publication Date**	Studies published more than 10 years ago; excludes outdated evidence not reflective of current lifestyle and dietary patterns

**Table 2:** Characteristics of the included studies.

Authors	Setting	Design	Sample & Sampling	Instrument	Outcomes	Intervention
[29]		Observational (cross-sectional)	Older adults with Alzheimer’s disease pathology	Not specified	Diet adherence associated with reduced AD pathology	Mediterranean–DASH intervention
[11]	USA	Randomised clinical trial (TRIUMPH)	Patients with resistant hypertension	Lifestyle modification programme	Reduced BP through lifestyle modification	Exercise, diet, behavioural changes
[29]	USA	Review/observational follow-up	African American men in barbershops	Community-based intervention	Sustained BP reduction	Barbershop-based BP management
[30]	Europe	Clinical trial	Hypertensive patients	Exercise testing	Assessed cardiovascular protective potential	Exercise testing intervention
[12]	Australia	Randomised trial	Hypertensive adults	Isometric handgrip training	Significant BP reduction	Handgrip resistance training
[16]	Nepal	Multicentre RCT	Hypertensive patients	Yoga intervention	Reduced BP after 3 months	Yoga programme
[31]	USA	Review	Older adults	Not specified	Guidance on hypertension management	Not applicable
[32]	USA	Registry (REVEAL)	Pulmonary hypertension patients	Clinical registry	5-year outcomes	Registry follow-up
[14]	Greece	Review	General population	Dietary evaluation	Salt restriction effective in BP reduction	DASH and Mediterranean diets
[27]	USA	Open-label intervention	Older adults in senior centres	Self-measured BP and meal intervention	Improved BP management	DASH-aligned congregate meals
[18]	USA	Intervention trial	Hypertensive patients	Lifestyle intervention programme	Reduced need for medication	Lifestyle interventions
[20]	USA	Cohort study	Type 2 diabetes patients	Aerobic fitness test	Higher aerobic fitness improved adherence to PA guidelines	Exercise interventions
[22]	Taiwan	RCT	Hypertensive patients	Web-based BP self-titration programme	Improved BP control	Digital intervention
[25]	Global	Review	General population	Epidemiological data	Physical inactivity linked to higher NCD burden	Not applicable
[33]	Iran	Observational study	Parkinson’s patients	Dietary survey	DASH diet protective against Parkinson’s	DASH diet
[26]	Portugal	Novel intervention trial	Elderly T2D patients	Exercise + dietary monitoring	BP management improved	Exercise in hypoxia + low-carb high-fat diet
[21]	Japan	Intervention study	Hypertensive patients	Walking intervention	Daily walking lowered BP	Walking programme
[34]	USA	Cross-sectional study	US adults	Dietary assessment	DASH/Mediterranean diet linked to metabolic health	Dietary evaluation
[13]	USA [17]	Cohort study	Physicians’ health study participants	Dietary pattern analysis	Reduced mortality with DASH/Med diets	Dietary patterns
[17]	India	Cluster RCT	Rural population	BP management strategies	Different strategies effective for CVD risk reduction	BP management interventions
[23]	Poland	Narrative review	Adults	Dietary and sleep review	Dietary adherence improved sleep quality	DASH, Mediterranean, MIND diets
[24]	Multinational	Cohort study	Haemodialysis patients	Dietary surveys	Diet adherence linked to reduced mortality	DASH and Mediterranean diets
[35]	Iran	Cross-sectional study	Adult women	Dietary surveys	DASH and Med diets differently associated with inflammatory markers	Dietary interventions
[2]	LMICs	Review	Populations in LMICs	Epidemiological data	Hypertension burden in LMICs	Not applicable
[36]	USA	RCT	Hypertensive adults	Exercise and weight loss programme	Exercise and weight loss reduced depressive symptoms	Exercise and weight loss
[37]	Canada	Review	Resistant hypertension patients	Not specified	Recommendations for resistant hypertension control	Not applicable
[38]	Australia	Observational study	Pregnant women	Dietary assessment	Adherence to diets reduced CRP levels	DASH and Mediterranean diets
[15]	Kenya	Cluster RCT	Patients with diabetes/hypertension	Group medical visits + microfinance	Improved management of hypertension and diabetes	Medical visits + microfinance
[39]	China	Cross-sectional study	Hypertensive patients in rural areas	Health literacy and self-management survey	Higher literacy improved QoL	Health literacy and self-management
[6]	China	RCT	Stage 1 hypertensive patients	Dietary intervention (DASH ± TRE)	DASH with TRE improved BP management	DASH diet + time-restricted eating

## Data Availability

No new data were created or analyzed in this study. Data sharing is not applicable to this article.

## Abbreviations

The following abbreviations are used in this manuscript:

DASH	Dietary Approach to Stop Hypertension
LMICs	Low- and middle-income countries
NCDs	Non-communicable diseases
PRISMA	Preferred Reporting Items for Systematic Reviews and Meta-anal
